# Elongation factor eEF1B modulates functions of the release factors eRF1 and eRF3 and the efficiency of translation termination in yeast

**DOI:** 10.1186/1471-2199-10-60

**Published:** 2009-06-22

**Authors:** Igor A Valouev, Gleb V Fominov, Elizaveta E Sokolova, Vladimir N Smirnov, Michael D Ter-Avanesyan

**Affiliations:** 1 Cardiology Research Center, 121552 Moscow, Russia

## Abstract

**Background:**

Termination of translation in eukaryotes is controlled by two interacting polypeptide chain release factors, eRF1 and eRF3. While eRF1 recognizes nonsense codons, eRF3 facilitates polypeptide chain release from the ribosome in a GTP-dependent manner. Besides termination, both release factors have essential, but poorly characterized functions outside of translation.

**Results:**

To characterize further the functions of yeast eRF1 and eRF3, a genetic screen for their novel partner proteins was performed. As a result, the genes for γ (*TEF4 *and *TEF3/CAM1*) and α (*TEF5*/*EFB1*) subunits of the translation elongation factor eEF1B, known to catalyze the exchange of bound GDP for GTP on eEF1A, were revealed. These genes act as dosage suppressors of a synthetic growth defect caused by some mutations in the *SUP45 *and *SUP35 *genes encoding eRF1 and eRF3, respectively. Extra copies of *TEF5 *and *TEF3 *can also suppress the temperature sensitivity of some *sup45 *and *sup35 *mutants and reduce nonsense codon readthrough caused by these omnipotent suppressors. Besides, overproduction of eEF1Bα reduces nonsense codon readthrough in the strain carrying suppressor tRNA. Such effects were not shown for extra copies of *TEF2*, which encodes eEF1A, thus indicating that they were not due to eEF1A activation.

**Conclusion:**

The data obtained demonstrate involvement of the translation elongation factor eEF1B in modulating the functions of translation termination factors and suggest its possible role in GDP for GTP exchange on eRF3.

## Background

Termination of translation of mRNA is governed by stop codons in the ribosomal A-site and polypeptide chain release factors of two classes. Class I release factors RF1 and RF2 in bacteria recognize UAA/UAG and UAA/UGA stop codons, respectively, whereas eukaryotes employ only one such factor, eRF1, which is able to decode all three nonsense codons [1]. The release factors of class I bind to the ribosomal A site, recognize the stop codon, and promote hydrolysis of the P-site peptidyl-tRNA to release completed polypeptide chain from the ribosome. Translation termination is stimulated by class II release factors, RF3 in bacteria, and eRF3 in eukaryotes. Both class II factors are GTPases enhancing the termination efficiency by stimulating activity of class I release factors in a GTP-dependent manner [2-4].

Determination of the crystal structure of human eRF1 has shown that it is composed of three domains and resembles by overall shape and dimensions a tRNA molecule with the N-terminal and middle domains corresponding to the tRNA's anticodon stem and aminoacyl acceptor stem, respectively [5]. eRF3 also has a complex structure and can be divided into at least two regions: a non-conserved N-terminal region and a conserved C-terminal region (domain C), showing a considerable sequence similarity to the translation elongation factor eEF1A, which brings aminoacyl-tRNAs to the ribosomal A site [6,7]. In the yeast *Saccharomyces cerevisiae *eRF1 and eRF3 are encoded by the essential *SUP45 *and *SUP35 *genes.

According to recent data, eRF3 functions in termination by applying its GTPase activity to assist eRF1 with stop codon recognition and ensures efficient hydrolysis of peptidyl tRNA [8,9]. Binding of eRF1 and eRF3·GTP to the pretermination ribosome forms a complex that is not active in peptide release, and further rearrangement, induced by GTP hydrolysis, is required for proper positioning of the GGQ loop of eRF1 in the peptidyl transferase center and triggering peptidyl-tRNA hydrolysis [9,10].

It is known that bacterial ribosomes can accelerate GDP for GTP exchange on RF3 [11]. In contrast, 80S ribosomes do not noticeably influence either binding of guanine nucleotides to the eRF1·eRF3 complex or GDP for GTP exchange on it [12]. *In vitro *studies have shown that while free eRF3 binds GDP, it binds GTP only in the presence of eRF1 [12-14]. However, kinetic analysis of interaction of eRF3 with guanine nucleotides demonstrated that eRF1 does not act like a classical guanine nucleotide exchange factor (GEF), which increases the dissociation of GDP from a GTPase, but rather as a GTP dissociation inhibitor for eRF3, promoting efficient ribosomal recruitment of its GTP-bound form [12].

Importantly, our knowledge of the mechanism of translation termination is mostly based on *in vitro *studies. However, genetic approaches allowed to reveal new molecular partners of both eRF1 and eRF3 in yeast [15-18] as well, as their functions unrelated to translation termination [17,19,20]. Here, we present results of the search for additional proteins functionally interacting with yeast eRF1 and eRF3. We found that extra copies of genes which encode the γ (*TEF3/CAM1 *and *TEF4*) and α (*TEF5*/*EFB1*) subunits of the translation elongation factor eEF1B, known to catalyze the exchange of bound GDP for GTP on eEF1A, suppressed synthetic lethal interaction between some mutant *SUP45 *and *SUP35 *alleles. Besides, extra copies of *TEF3 *and *TEF5 *relieved temperature sensitivity of some mutants in these genes and reduced nonsense readthrough in one of the *sup45 *mutants. Overproduction of eEF1Bα also reduced nonsense readthrough in the *sup35 *mutant and in the strain carrying ochre suppressor tRNA. The described effects most likely were not mediated by an increase of eEF1A activity. Obtained results allowed us to suggest that the observed effects are mediated by the ability of yeast eEF1B to stimulate guanine-nucleotide exchange on eRF3.

## Methods

### Strains, media, growth conditions and genetic methods

Yeast strains were grown in either YEPD (1% Bacto yeast extract, 2% peptone, 2% dextrose) or defined synthetic complete media (C or C-) supplemented with 2% dextrose as a carbon source. The 5-fluoroorotic acid (5FOA) medium was prepared as described [21]. The final concentration of 5FOA was 0.9 mg/ml. The expression of the *tetO*_2_-controlled *SUP45 *was repressed by incubation of corresponding strains on medium selective for the pCM183-SUP45 plasmid which contained 20 μg/ml doxycycline. LB and 2× YT media were used for bacteria [22]. Appropriate amounts of antibiotics, amino acids, and bases were added when necessary. Yeast cells were grown at 30°C, if not indicated otherwise, and bacteria at 37°C. DNA transformation of lithium acetate-treated yeast cells was performed as described previously [23]. *Escherichia coli *cells were transformed by the method described in [24]. *E. coli *strain DH5α [*supE*44 Δ*lac *U169 (φ 80 *lacZΔ*M15) *hsdR*17 *recA*1 *endA*1 *gyrA*96 *thi*-1 *relA*1] was used in cloning experiments [22]. The yeast strains used are listed along with their genotypes in Table 1. To construct the strain BY4741-ΔSUP35, the *SUP35 *gene was deleted in the strain BY4741, with the use of the *Eco*32I-*Not*I fragment of pBSS35::H3 as a disruption cassette. PCR was used to prove correct integration of the disruption cassettes.

**Table 1:** *S. cerevisiae *strains

Strain	Genotype	Source
33G-D373	*MATα ura3-52 leu2-3,112 trp1-289 his7-1 lys9-A21 ade2-144,717 pheA-11*	[25]
33G-D373-rSL23	*MATα ura3-52 leu2-3,112 trp1-289 his7-1 lys9-A21 ade2-144,717 pheA-11 sup45-sl23*^*ts*^	[20]
33G-D373-r36	*MATα ura3-52 leu2-3,112 trp1-289 his7-1 lys9-A21 ade2-144,717 pheA-11 sup45-36*^*ts*^	[20]
33G-D373-rSL23-r35C	*MATα ura3-52 leu2-3,112 trp1-289 his7-1 lys9-A21 ade2-144,717 pheA-11 sup45-sl23*^*ts*^*SUP35-C*	[20]
33G-D373-r36-r35C	*MATα ura3-52 leu2-3,112 trp1-289 his7-1 lys9-A21 ade2-144,717 pheA-11 sup45-36*^*ts*^*SUP35-C*	[20]
8V-H80	*MATα ade1-14 his7-1 leu2-3,112 ura3-52 trp1-289 lys2-A12*	This work
8V-H80-168	*MATα ade1-14 his7-1 leu2-3,112 ura3-52 trp1-289 lys2-A12 sup35-168*	This work
8V-H80-196	*MATα ade1-14 his7-1 leu2-3,112 ura3-52 trp1-289 lys2-A12 sup35-196*	This work
BY4741	*MAT*a *his3Δ1 leu2Δ0 met15Δ0 ura3Δ0*	Open Biosystems
BY4741-ΔSUP35	*MAT*a *his3Δ1 leu2Δ0 met15Δ0 ura3Δ0 sup35::HIS3 *[pRG416-SUP35C]	This work
50V-H78	*MATα ade1-14 ura3-52 leu2-3,112 his3-D200 his5-2 lys1-1 met8-1 ilv1-1 SUP4*	This work

Plasmid that supports viability of the BY4741-ΔSUP35 strain is indicated in square brackets.

### Plasmids and nucleic acid manipulation

All DNA manipulations were carried out by standard protocols [22]. The plasmids used are listed along with their essential characteristics in Table 2. The plasmid YEplac195-SUP45 was constructed by inserting the *SUP45*-containing *Xba*I-*Pvu*II fragment of the pRG415-SUP45 plasmid into the *Xba*I and *Sma*I sites of YEplac195. The mutant alleles of *SUP35*, namely *sup35-168*^*ts *^and *sup35-196*^*ts*^, were amplified by PCR (primers SUP35prod and SUP35terr; Table 3) using genomic DNA of the strains 8V-H80-168 (*sup35-168*^*ts*^) and 8V-H80-196 (*sup35-196*^*ts*^) as a template. The amplification products were digested with *Xba*I and *Sac*I and inserted into the same sites of pRS315, thus creating the pRS315-sup35-168 or pRS315-sup35-196 plasmids. The plasmid mutant alleles were sequenced. To construct YEplac181-SUP35C, the *Eco*RI-*Xba*I fragment carrying *SUP35-C *of the pRS315-SUP35C plasmid [20] was inserted between the same sites of YEplac181.

**Table 2:** Plasmids

Plasmid	Characteristics	Source
pCM183-SUP45	Centromeric *TRP1 *vector containing *SUP45 *under the control of regulatable *tetO*_2 _promoter	[20]
YEplac195	Multicopy *URA3 *vector	[26]
YEplac195-SUP45	Same as YEplac195, but with *SUP45*	This work
YEplac195-TEF2	Same as YEplac195, but with *TEF2*	This work
YEplac195-TEF4	Same as YEplac195, but with wild type *TEF4*	This work
YEplac195-TEF5	Same as YEplac195, but with wild type *TEF5*	This work
YEplac195-TEF5Δi	Same as YEplac195, but with *TEF5 *without intron (*TEF5-Δi*)	This work
YEplac181	Multicopy *LEU2 *vector	[26]
YEplac181-TEF2	Same as YEplac181, but with *TEF2*	This work
YEplac181-TEF5Δi	Same as YEplac181, but with *TEF5 *without intron (*TEF5-Δi*)	This work
YEplac181-TEF3	Same as YEplac181, but with *TEF3*	This work
YEplac181-TEF4Δi	Same as YEplac181, but with *TEF4 *without intron (*TEF4-Δi*)	This work
YEplac181-SUP35C	Same as YEplac181, but with *SUP35-C*	This work
pRS315	Centromeric *LEU2 *vector	[27]
pRS315-SUP35	Same as pRS315, but with *SUP35*	[20]
pRS315-sup35-168	Same as pRS315, but with the *sup35-168*^*ts *^allele	This work
pRS315-sup35-196	Same as pRS315, but with the *sup35-196*^*ts *^allele	This work
pRS315-SUP45	Same as pRS315, but with *SUP45*	[17]
pRS315-sup45-sl23	Same as pRS315, but with the *sup45-sl23*^*ts *^allele	[17]
pRG416-SUP35C	Centromeric *URA3 *vector containing *SUP35-C*	[7]
pRG415-SUP45	Centromeric *URA3 *vector containing *SUP45*	[19]
pBSS35::H3	Bacterial vector containing the *sup35::HIS3 *disruption allele	This work

**Table 3:** Oligonucleotides

Oligonucleotide	Sequence
EFB1E1rev	5'-CCTTCAATGTATGACTTGT-3'
EFB1E2dir	5'-TACTGCTGTTTCTCAAGCTGA-3'
TEF4E1r	5'-ATAAAATTGGATAGCCAAAGC-3'
TEF4E2d	5'-GTGGCTAATCAAGTTGCCGA-3'
CAM1r	5'-CTGCTCTGCTCAACGGAA-3'
CAM1d	5'-TGCTCTAGACGGGCTGATACGGCCATT-3'
TEF2r	5'-GAGGCCGTCTTTTGTTGA-3'
TEF2d	5'-CGTGGATCCTAGGCGCTTCCCCTGCCG-3'
SUP35prod	5'-ACGAGCTCAAATTATTATTTTTTACTAAG-3'
SUP35terr	5'-AATTCTAGATATATTGAGAGGTGA-3'

To obtain a yeast genomic library, chromosomal DNA of the 5V-H19 strain [28] was partially digested with *Sau*3A, fractionated on agarose gel and DNA fragments ranging from 4 to 12 kb were isolated. The ends of chromosomal DNA fragments were partially filled in with Klenow enzyme and ligated to the partially filled in *Sal*I site of the YEplac195 plasmid. A plasmid with the 4.5 kb chromosomal DNA fragment containing *TEF4 *and *RRP14 *was isolated from the genomic library. The *Eco*RI-*Sma*I *TEF4-*containing fragment of this plasmid was inserted into the same sites of YEplac195, thus resulting in YEplac195-TEF4. The intronless variant of *TEF4 *(*TEF4-Δi*) was obtained as follows. The *TEF4*-containing *Pst*I-*Eco*RI DNA fragment of YEplac195-TEF4 was inserted between the same sites of pUC18 [29]. The obtained plasmid was used as a template for PCR with the primers TEF4E1r and TEF4E2d (Table 3). Amplified DNA fragment, representing the plasmid lacking *TEF4 *intron sequence, was self ligated, resulting in the plasmid bearing *TEF4-Δi*. The *TEF4-Δi*-containing *Pst*I-*Eco*RI DNA fragment of this plasmid was inserted between the same sites of YEplac181 resulting in the plasmid YEplac181-TEF4Δi.

The *TEF3 *gene was amplified by PCR (primers CAM1r and CAM1d; Table 3) using genomic DNA of the strain 33G-D373 as a template. The amplification product was digested with *Xba*I and inserted into the *Xba*I and *Sma*I sites of YEplac181, thus generating YEplac181-TEF3. The *TEF5 *gene was amplified by PCR (primers EFB1r and EFB1d; Table 3) using genomic DNA of 33G-D373 as a template. The amplification product was digested with *Bam*HI and inserted into the *Bam*HI and *Ecl*136II sites of YEplac195, thus generating YEplac195-TEF5. The intronless variant of *TEF5 *(*TEF5-Δi*) was obtained as follows. The *TEF5*-containing *Bam*HI-*Sac*I fragment of YEplac195-TEF5 was inserted between the same sites of the pUC18 plasmid [29]. The obtained plasmid was used as a template for PCR with primers EFB1E1rev and EFB1E2dir (Table 3). Amplified DNA fragment with *TEF5 *lacking intron sequence, was self ligated to obtain the plasmid bearing *TEF5-Δi*. The *TEF5-Δi*-containing *Bam*HI-*Ecl*136II DNA fragment of this plasmid was inserted between the same sites of YEplac181 and YEplac195, thus resulting in the plasmids YEplac181-TEF5Δi and YEplac195-TEF5Δi, respectively. The presence of the multicopy plasmid YEplac181-TEF5Δi in yeast cells caused approximately 6-fold overproduction of eEF1Bα, as was shown by probing appropriately diluted cell lysates with the mouse polyclonal antibody against eEF1Bα. The *TEF2 *gene was amplified by PCR (primers TEF2r and TEF2d; Table 3) using genomic DNA of 33G-D373 as a template. The amplification product was digested with *Bam*HI and inserted into the *Bam*HI and *Ecl*136II sites of YEplac195, thus generating YEplac195-TEF2. The plasmid YEplac181-TEF2 was constructed by inserting the *Bam*HI-*Sna*BI fragment of YEplac195-TEF2 containing *TEF2 *into the same sites of YEplac181. To construct the *SUP35 *disruption cassette (plasmid pBSS35::H3), the *Eco*105I-*Mun*I internal fragment of the *SUP35 *gene was replaced with the *HIS3 *selectable marker.

### Determination of the efficiency of nonsense codon readthrough

Following plasmids carrying tandem *Renilla *and firefly luciferase genes separated by a single in-frame stop codon or a corresponding sense codon control were used to measure the efficiency of nonsense codon readthrough: pDB691 (UGAC), pDB690 (CGAC), pDB723 (UAAC), pDB722 (CAAC), pDB720 (UAGC) and pDB721 (CAGC) [30]. Luciferase assays were performed with a dual luciferase reporter assay system (Promega). Assays were performed as described [31] with minimal modifications using Glomax 20/20 luminometer (Promega). Assays were done in triplicate, and the data are expressed as the means ± the standard errors. The percent readthrough in each strain is expressed as the ratio of *Renilla *luciferase activity/firefly luciferase activity (nonsense) divided by the ratio of *Renilla *luciferase activity/firefly luciferase activity (sense) multiplied by 100. For other details, see [32].

## Results

### TEF3, TEF4 and TEF5 extra copies suppress synthetic lethal interaction between the *sup45-sl23*^*ts *^and *SUP35-C *mutant alleles

**Figure 1:**
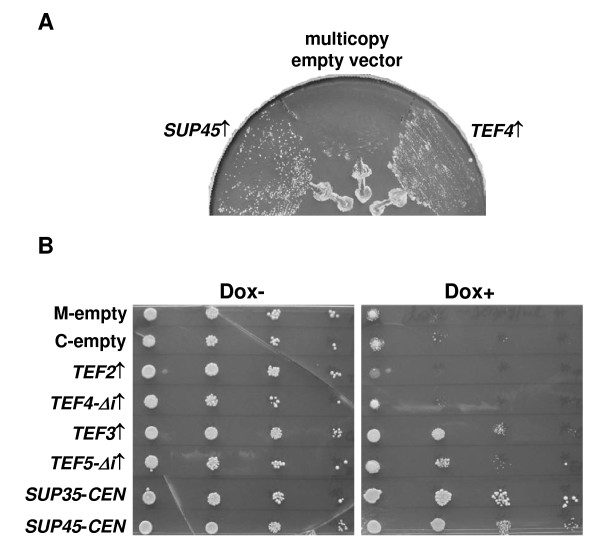
**Extra copies of *TEF3*, *TEF4 *and *TEF5 *suppress synthetic lethality between *sup45-sl23*^*ts*^and *SUP35-C***. (A) The multicopy plasmids YEplac195-TEF4 (*TEF4*↑), YEplac195-SUP45 (*SUP45*↑), with the *TEF4 *and *SUP45 *genes, respectively, or the multicopy empty vector YEplac195 were introduced into the strain 33G-D373-rSL23-r35C with the *sup45-sl23*^*ts *^and *SUP35-C *alleles, bearing the centromeric plasmid pCM183-SUP45. Obtained transformants were streaked on C-Ura medium containing doxycycline and incubated at 30°C for 2 days. (B) Transformants of the strain 33G-D373-rSL23-r35C containing the pCM183-SUP45 plasmid along with the empty multicopy YEplac181 (M-empty) or centromeric pRS315 (C-empty) vectors were used as negative control. Transformants with the centromeric plasmids pRS315-SUP35C (*SUP35-CEN*) and pRS315-SUP45 (*SUP45-CEN*) bearing the *SUP35 *and *SUP45 *genes, respectively, represented positive control, since a single copy of either one of these genes abolished synthetic lethality [20]. Growth of the control transformants was compared with that of the transformants bearing one of the multicopy plasmids YEplac181-TEF2 (*TEF2↑*), YEplac181-TEF4Δi (*TEF4-Δi↑*), YEplac181-TEF3 (*TEF3↑*) or YEplac181-TEF5Δi (*TEF5-Δi↑*). Transformants were grown in liquid C-Leu medium at 30°C and diluted to an OD_600 _of 1.0. Ten-fold serial dilutions were spotted onto C-Leu medium which contained (Dox+) or did not contain (Dox-) doxycycline and grown at 30°C for 3 days.

Earlier, we have identified mutations in the *SUP45 *gene which manifest lethality in combination with the *SUP35-C *allele, which encodes eRF3 lacking the inessential N-terminal region. The study of these mutations and their lethal interaction with *SUP35-C *indicated the role of N-terminal region of yeast eRF3 in uncharacterized non-translational function of the eRF1·eRF3 termination complex [20]. To clarify the non-translational mechanisms associated with the eRF1·eRF3 complex we performed a screen for dosage suppressors of this synthetic lethality. To isolate such suppressors, we used the strain 33G-D373-rSL23-r35C which carried the *sup45-sl23*^*ts *^mutation and the *SUP35-C *allele, as well as the centromeric pCM183-SUP45 plasmid with the *TRP1 *selectable marker and *SUP45 *under the control of regulatable *tetO*_2 _promoter [20]. This strain, which was unable to grow on medium containing 20 μg/ml doxycycline due to repression of the plasmid wild type *SUP45 *gene, was transformed with a *S. cerevisiae *genomic library based on the multicopy plasmid YEplac195, carrying the *URA3 *selectable marker. The transformants were selected on uracil omission medium, which contained doxycycline. From about one hundred of transformants selected only two were unable to grow on medium containing doxycycline after the loss of the library plasmid on 5FOA medium. Restriction analysis showed that these transformants contained plasmids with identical 4.5 kb inserts of genomic DNA. Both plasmids carried a DNA fragment with the *TEF4 *and *RRP14 *genes encoding a subunit of the translation elongation factor eEF1B [33] and the Rrp14 protein involved in ribosome synthesis and positioning of the mitotic spindle [34], respectively, but subsequent deletion analysis indicated that suppression required the presence of *TEF4 *alone (Figure 1A).

eEF1B serves as a GEF for eEF1A, accelerating dissociation of its complex with GDP. In contrast to mammals, in which eEF1B is composed of three subunits, α, β and γ, yeast eEF1B contains only two of them, α and γ. Yeast eEF1Bγ is encoded by two inessential genes, *TEF3 *and *TEF4 *[33,35], and probably serves as a positive regulator of the catalytic eEF1Bα subunit [36-38], which is encoded by a single essential gene *TEF5 *[39]. We isolated the genomic *TEF3 *and *TEF5 *genes to study the ability of their extra copies to suppress synthetic lethal interaction between *sup45-sl23*^*ts *^and *SUP35-C*. The *TEF4 *and *TEF5 *genes contain intervening sequences encoding the small nucleolar RNAs snR38 and snR18, respectively. The latter RNA was shown to be involved in fine tuning of translation termination in yeast [40]. To determine, whether the overproduction of the *TEF4*- and *TEF5*-encoded proteins themselves was responsible for suppression of synthetic lethality, we constructed multicopy plasmids carrying the *TEF4 *or *TEF5 *genes without introns (*TEF4-Δi *and *TEF5-Δi*, respectively). Surprisingly, a spot test did not reveal any noticeable suppressor effect for *TEF4-Δi *extra copies, while high dosage of *TEF3 *and *TEF5-Δi *suppressed synthetic lethality (Figure 1B). It is noteworthy that the absence of suppression for the *TEF4-Δi *extra copies was not due to the lack of an intron, since no suppression in a spot test was observed in transformants carrying the plasmid with wild type *TEF4 *(data not shown). This discrepancy indicates that *TEF4 *extra copies suppress synthetic lethality less efficiently than extra copies of either *TEF3 *or *TEF5*.

The *sup45-sl23*^*ts *^mutation is not the only mutation in the *SUP45 *gene manifesting synthetic lethality with *SUP35-C*; the same effect was found for the *sup45-36*^*ts *^mutation [20]. Both mutations altered the N domain of eRF1 and caused substitutions of amino acids located in proximity to each other, Ser30 to Phe replacement in the *sup45-sl23*^*ts *^mutant [20] and Leu34 to Ser in *sup45-36*^*ts *^.[41]. However, *TEF5-Δi *was unable to act as a dosage suppressor of the lethal interaction between *sup45-36*^*ts *^and *SUP35-C *indicating the *sup45 *allele specificity of the suppressor effect.

### Temperature sensitivity of *sup45 *and *sup35 *mutants can be suppressed by overproduction of eEF1B subunits

**Figure 2:**
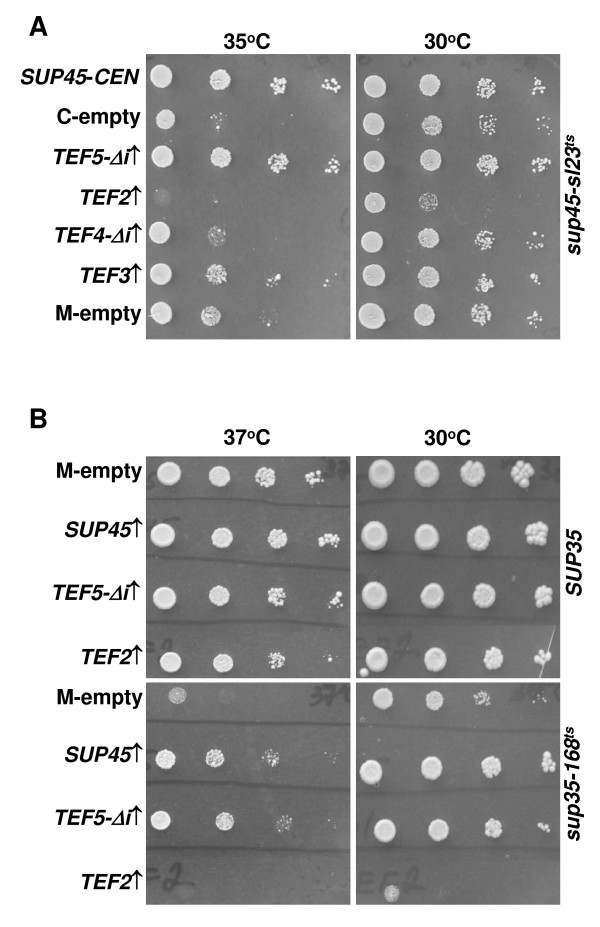
**Effects of extra copies of the *TEF3*, *TEF4 *and *TEF5 *genes on temperature sensitivity of the *sup45-sl23*^*ts *^and *sup35-168*^*ts *^mutants**. (A) Growth of transformants of the 33G-D373-sl23 strain with the *sup45-sl23*^*ts *^mutation, bearing the centromeric or multicopy empty vectors pRS315 (C-empty) or YEplac181 (M-empty), respectively (negative controls), or centromeric plasmid pRS315-SUP45 (*SUP45-CEN*) (positive control), was compared with growth of transformants, bearing one of the multicopy plasmids: YEplac181-TEF2 (*TEF2↑*), YEplac181-TEF3 (*TEF3↑*), YEplac181-TEF4Δi (*TEF4-Δi↑*) or YEplac181-TEF5Δi (*TEF5-Δi↑*). Transformants were grown as described in legend to Figure 1B, except cell suspensions were spotted on C-Leu without doxycycline and incubated at 30°C or 35°C. (B) The strain BY4741-ΔSUP35 with disrupted *SUP35 *contained the *URA3 SUP35-C *centromeric plasmid pRG416-SUP35C, which was shuffled for either the centromeric *LEU2 *pRS315-SUP35 or pRS315-sup35-168 plasmids bearing the wild type *SUP35 *or mutant *sup35*-*168*^*ts *^alleles, respectively. Shuffling for the *sup35*-*168*^*ts *^plasmid produced transformants unable to grow at 37°C. Then, the strains with either the *SUP35 *or *sup35*-*168*^*ts *^plasmids were transformed with one of the multicopy plasmids YEplac195 (M-empty), YEplac195-TEF2 (*TEF2↑*), YEplac195-TEF5Δi (*TEF5-Δi↑*) or YEplac195-SUP45 (*SUP45↑*). The transformants were grown in liquid C-Ura medium and diluted to OD_600 _of 1.0. Ten-fold serial dilutions were spotted onto C-Ura medium and grown at 30°C or 37°C for 4 days.

The data obtained indicated a role of eEF1B in functioning of the eRF1·eRF3 complex. This raised a question, whether eEF1B overproduction can suppress mutational defects of the individual components of this complex? To address this question, we examined, if temperature sensitivity of the *sup45-sl23*^*ts *^and *sup45-36*^*ts *^mutants and some *sup35*^*ts *^mutants can be alleviated by the identified dosage suppressors. Efficient suppression of temperature sensitivity of the strain 33G-D373-rSL23, carrying the *sup45-sl23*^*ts *^mutation, was revealed only for extra copies of the *TEF5-Δi *gene. Extra copies of the *TEF3 *but not of the *TEF4 *gene caused weak but reproducible alleviation of the *sup45-sl23*^*ts *^temperature sensitivity (Figure 2A). This difference in the suppression efficiency can be expected, since eEFB1γ, encoded by *TEF3*, is a regulatory subunit which stimulates activity of the *TEF5*-encoded eEFB1α [36-38]. Importantly, extra copies of *TEF5-Δi *did not suppress temperature sensitivity of the *sup45-36*^*ts *^mutant 33G-D373-r36, which agrees with the *sup45 *allele specific suppression of synthetic lethality (data not shown).

Since *TEF5 *was found to be the most efficient dosage suppressor of temperature sensitivity of the *sup45-sl23*^*ts *^mutant, effects of its extra copies on growth of *sup35 *mutants were studied further. Examination of 21 *sup35*^*ts *^mutants of the strain 8V-H80 revealed two mutants with phenotypes that depended on the presence of the *TEF5-Δi *multicopy plasmid: temperature sensitivity of the *sup35*-*168*^*ts *^mutant was suppressed, while growth of the *sup35-196*^*ts *^was inhibited even at permissive temperature (data not shown). The ability of *TEF5-Δi *extra copies to suppress temperature sensitivity of *sup35*-*168*^*ts *^was reproduced in the BY4741-ΔSUP35 strain. Interestingly, extra copies of *SUP45 *also alleviated temperature sensitivity of the *sup35*-*168*^*ts *^mutant (Figure 2B). Similar effect of *SUP45 *overdose was earlier shown for other *sup35*^*ts *^mutants [42].

Sequencing of the *sup35*^*ts *^mutant alleles, manifestation of which depended on *TEF5-Δi *extra copies, revealed that the mutations altered the C-terminal region of eRF3 located downstream of its GTP-binding domain: *sup35*-*168*^*ts *^caused replacement of Leu553 to Pro, while nucleotide substitution in the *sup35-196*^*ts *^allele corresponded to change of Thr667 to Pro.

### Extra copies of *TEF5 *and *TEF3 *reduce efficiency of nonsense codon readthrough

**Figure 3:**
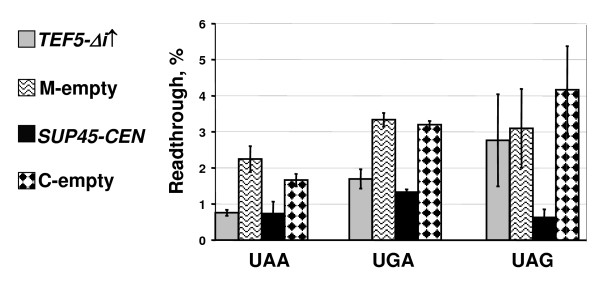
**High dosage of *TEF5 *reduces UAA and UGA readthrough levels in the *sup45-sl23*^*ts *^mutant**. The 33G-D373-rSL23 strain with the *sup45-sl23*^*ts *^mutation was transformed with one of the following plasmids: multicopy plasmids YEplac181-TEF5Δi (*TEF5-Δi↑*) or YEplac181 (M-empty), and centromeric plasmids pRS315-SUP45 (*SUP45-CEN*) or pRS315 (C-empty). The appropriate dual luciferase reporter plasmids were introduced into cells of obtained transformants. The transformants carrying plasmid pairs were grown in liquid C-Leu, Ura medium at 30°C and then readthrough levels were measured as described in Methods. The plasmid YEplac181-TEF5Δi causes a statistically significant decrease of UAA and UGA readthrough levels (*P *≤ 0.02 and *P *≤ 0.01, respectively) compared to those in transformants carrying YEplac181. Comparison of transformants bearing pRS315-SUP45 and pRS315 revealed statistically significant difference in readthrough of all stop codons: *P *≤ 0.1, *P *≤ 0.001 and *P *≤ 0.05 for UAA, UGA and UAG codons, respectively.

**Figure 4:**
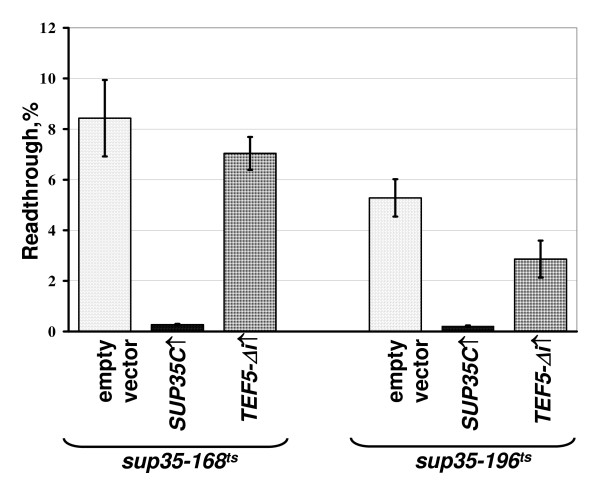
**High dosage of *TEF5 *reduces efficiency of UAA readthrough in the *sup35 *mutants**. The strains 8V-H80-168 (*sup35-168*^*ts*^) and 8V-H80-196 (*sup35-196*^*ts*^) were transformed with one of the multicopy plasmids YEplac181-TEF5Δi (*TEF5-Δi↑*), YEplac181-SUP35C (*SUP35C↑*) or YEplac181 (empty vector) and then with the appropriate dual luciferase reporter plasmids. The transformants with plasmid pairs were grown and readthrough measured as described in legend to Figure 3 and in Methods. The difference in UAA readthrough in the mutant *sup35-196*^*ts *^strain with the YEplac181-TEF5Δi and YEplac181 plasmids is statistically significant (*P *≤ 0.1).

Genetic interactions described above show that eEF1B is functionally related to the yeast release factors, but do not indicate its involvement in translation termination. To test the latter possibility we investigated the influence of a high dosage of the genes for eEF1B subunits on nonsense readthrough in the *sup45 *and *sup35 *omnipotent suppressor mutants. Extra copies of *TEF5-Δi *decreased the levels of UAA и UGA readthrough in the *sup45-sl23*^*ts *^mutant almost to the levels observed in this strain in the presence of wild type *SUP45*, but did not affect readthrough of UAG (Figure 3). A smaller decrease of UAA readthrough due to eEF1Bα overproduction was found in the strain carrying the *sup35-196*^*ts *^mutation, though this effect was statistically insignificant in the *sup35*-*168*^*ts *^mutant (Figure 4). The observed effects agree with the fact that extra copies of *TEF5 *weakened suppression of the *his7-1 *UAA mutation in the strain with the *sup35-25 *omnipotent suppressor [43]. However, other results demonstrated that overproduction of eEF1Bα could increase the levels of nonsense readthrough in strains lacking suppressor mutations [44,45].

**Figure 5:**
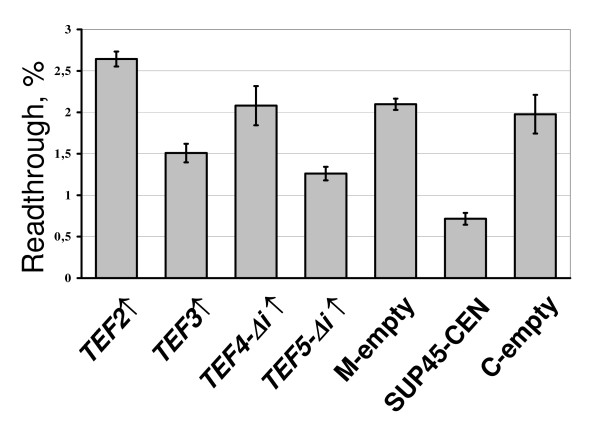
**Extra copies of *TEF3*, but not of *TEF4 *reduce efficiency of UAA readthrough in the *sup45-sl23*^*ts *^mutant**. The 33G-D373-rSL23 strain was transformed with one of the multicopy plasmids YEplac181-TEF2 (*TEF2↑*), YEplac181-TEF3 (*TEF3↑*), YEplac181-TEF4Δi (*TEF4-Δi↑*), YEplac181-TEF5Δi (*TEF5-Δi↑*), YEplac181 (M-empty) or centromeric plasmids pRS315-SUP45 (*SUP45-CEN*) or pRS315 (C-empty). The appropriate dual luciferase reporter plasmids were then introduced into cells of obtained transformants. The transformants with plasmid pairs were grown and readthrough measured as described in legend to Figure 3 and in Methods. The plasmids YEplac181-TEF3, YEplac181-TEF5Δi and pRS315-SUP45 cause a statistically significant (*P *≤ 0.02, *P *≤ 0.002 and *P *≤ 0.01, respectively) decrease of UAA readthrough levels compared to those in transformants carrying corresponding empty vectors. The levels of UAA readthrough are increased in transformants with YEplac181-TEF2 as compared with that in transformants with YEplac181 (*P *≤ 0.01).

**Figure 6:**
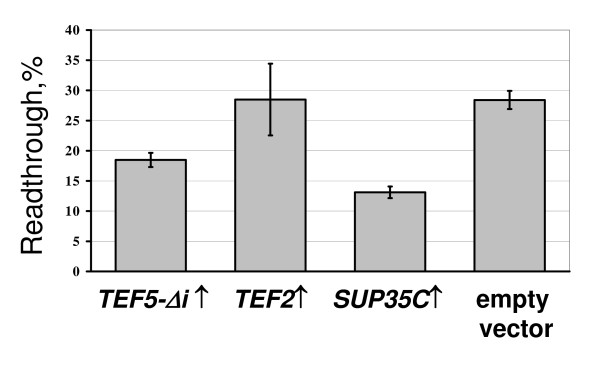
**Extra copies of *TEF5 *manifest an antisuppressor effect in the strain with the ochre tRNA *SUP4 *suppressor**. The strain 50V-H78 (*SUP4*), was transformed with one of the multicopy plasmids YEplac181-TEF5Δi (*TEF5-Δi↑*), YEplac181-TEF2 (*TEF2↑*), YEplac181-SUP35C (*SUP35C↑*) or YEplac181 (empty vector). The appropriate dual luciferase reporter plasmids were then introduced into cells of obtained transformants. The transformants with plasmid pairs were grown and readthrough measured as described in legend to Figure 3 and in Methods. The plasmids YEplac181-TEF5Δi and YEplac181-SUP35C cause a statistically significant (*P *≤ 0.01 and *P *≤ 0.002, respectively) decrease of UAA readthrough levels compared to those in transformants carrying YEplac181.

In addition to *TEF5-Δi*, a decrease of UAA readthrough in the *sup45-sl23*^*ts *^mutant was also observed for a high dosage of *TEF3*, but not of *TEF4-Δi *(Figure 5). At last, overproduction of eEF1Bα manifested an antisuppressor effect not only in the *sup35 *and *sup45 *suppressor mutants, since it reduced the levels of nonsense readthrough caused by the ochre tRNA suppressor *SUP4 *in the strain 50V-H78. Remarkably, *SUP35-C *overdose caused in this strain just a slightly stronger antisuppressor effect than extra copies of *TEF5-Δi *(Figure 6).

### Effects of eEF1B overproduction are not mediated by an increase of eEF1A activity

Translation elongation factor eEF1A is a classic G protein involved in the GTP-dependent binding of amino-acylated tRNA, and the eEF1B α subunit, associated with its γ subunit, functions as a GEF for eEF1A [46,47]. This suggests that the effects of overproduction of eEF1B subunits, observed in our work, are mediated by an increased activity of its target protein, eEF1A. However, extra copies of *TEF2*, one of the two yeast genes encoding eEF1A, neither suppressed synthetic lethality between *sup45-sl23*^*ts *^and *SUP35-C *(Figure 1), nor alleviated temperature sensitivity of the *sup35*-*168*^*ts *^or *sup45-sl23*^*ts *^mutants (Figure 2), thus ruling out this possibility. In contrast, extra copies of *TEF2 *could even inhibit growth of the *sup35*-*168*^*ts *^and *sup45-sl23*^*ts *^mutants, though in these experiments growth inhibition was not observed when the strains expressed wild type *SUP45 *(Figure 1B, Dox- panel) or *SUP35 *(Figure 2B).

Measurement of nonsense readthrough levels confirmed this conclusion: in contrast to *TEF5-Δi *and *TEF3*, the overdose of which caused an antisuppressor effect, extra copies of *TEF2 *either did not reduce UAA readthrough in the *SUP4 *ochre suppressor-carrying strain (Figure 6), or even stimulated it in the *sup45-sl23*^*ts *^mutant (Figure 5). It is also necessary to stress that in contrast to *TEF2*, extra copies of *SUP35-C *caused a decrease of UAA readthrough in the *SUP4 *strain (Figure 6).

## Discussion

In eukaryotes a GTP-bound form of eRF3 is active in termination, because GTP hydrolysis by eRF3 on the ribosome is required for the efficient translation termination [2,4,8,9]. However, *in vivo *eRF3 should mostly be present in its GDP-bound form, since (i) when termination is completed it is released from the ribosome in a complex with GDP, and (ii) newly synthesized eRF3 has a high affinity to GDP, being able to bind GTP only upon interaction with eRF1 [12-14]. It is also important that though eRF1 is able to enhance the levels of the GTP-bound form of eRF3, it does not act like classic GEFs, which increase the dissociation of GDP from a GTPase [12]. Since no GEF was identified for eRF3, it could be that it is able to dissociate GDP spontaneously similarly to eEF1A which can do this, although with a 700-fold slower rate than with the assistance of eEFB1 [48]. However, despite this, the results of our work suggest that eEFB1 acts as a GEF for eRF3. This suggestion is based on (i) the functional interaction of the α and γ subunits of eEF1B with the release factors and (ii) the reduced nonsense readthrough caused by overproduction of eEF1B subunits. It is also necessary to stress that eEF1Bγ and eEF1Bα are structurally different proteins and the former can stimulate *in vitro *the nucleotide exchange activity of the latter [36-38]. This additionally relates the observed effects of their overproduction to the role of the eEF1B factor in GDP/GTP exchange on eRF3. It is noteworthy that the effects of an overdose of the *TEF3*- and *TEF4*-encoded γ subunit of eEF1B were different. While extra copies of either gene can suppress synthetic lethal interaction between the *sup45-sl23*^*ts *^and *SUP35-C *mutant alleles, only *TEF3 *extra copies reduce nonsense readthrough in the *sup45-sl23*^*ts *^mutant and alleviate its growth at restrictive temperature. This indicates a functional difference between *TEF3*- and *TEF4*-encoded proteins, which agrees with earlier findings [33]. It may be suggested that the complex of eEF1Bα with the *TEF3*-encoded protein preferentially stimulates guanine-nucleotide exchange on eRF3, while its complex with the *TEF4*-encoded eEF1Bγ has a preference for eEF1A.

Since eEF1B acts as GEF for eEF1A, its overproduction should increase the concentration of active (GTP-bound) eEF1A. However, it is unlikely that the effects of eEF1B overproduction observed in this work were mediated by activation of eEF1A, because extra copies of *TEF2 *did not suppress synthetic lethality between *sup45-sl23*^*ts *^and *SUP35-C*, as well as temperature sensitivity of the *sup35*-*168*^*ts *^and *sup45-sl23*^*ts *^mutants and even could inhibit growth of these mutants. It is known that, besides translation, overexpression of eEF1A in yeast also affects actin cytoskeleton which may be the cause of growth inhibition [45]. However, we observed that extra copies of *TEF2 *caused a noticeable growth inhibition only when mutant *sup35 *or *sup45 *alleles were expressed. Similarly to eEF1A, yeast eRF1 and eRF3 have nontranslational functions and their deficiency, as well as mutational inactivation, may inhibit yeast cell growth via perturbations of the cytoskeleton organization [17,19]. Therefore, one can suggest that *TEF2 *extra copies and *sup35 *or *sup45 *mutations act synergistically to affect cytoskeleton. It is also important that extra copies of *TEF2 *did not influence nonsense readthrough in the strain with the *SUP4 *tRNA ochre suppressor and even increased it in the *sup45-sl23*^*ts *^mutant. At the same time, in contrast to *TEF2*, extra copies of *SUP35-C*, encoding the C domain of eRF3 caused a decrease of UAA readthrough in the *SUP4 *strain, which also makes eRF3 an appropriate target for the eEF1B action.

It is noteworthy that an overproduction of eEF1Bα suppressed temperature sensitivity of the *sup35*-*168*^*ts *^mutant, but did not noticeably reduce nonsense codon readthrough indicating that temperature sensitivity of this mutant is not due to a defect of translation termination. This suggests that even slight stimulation of GDP for GTP exchange on eRF3, which did not restore translation termination in the *sup35*-*168*^*ts *^mutant, could repair defect of the non-translational function of mutant eRF3. Temperature sensitivity of *sup45-sl23*^*ts *^is also unrelated to a high level of nonsense readthrough [20], though in this mutant overproduction of eEF1Bα alleviated the growth defect and decreased UAA and UGA readthrough. Importantly, overproduction of eEF1Bα did not suppress temperature sensitivity of the *sup45-36*^*ts *^mutant. It was shown that at restrictive temperature the *sup45-36*^*ts *^mutation affects cytokinesis due to a defect of the non-translational function of eRF1 mediated by its interaction with the myosin light chain Mlc1. Since the eRF1·Mlc1 complex, does not contain eRF3 [17], it is logical that temperature sensitivity of this mutant did not depend on the levels of eEF1Bα.

Taken together, these data suggest that the guanine-nucleotide exchange activity of eEF1B plays a role in the functioning of release factors in translation termination as well as in their functions outside of termination. Though the mechanisms which underlie suppression of mutational defects of eRF1 and eRF3 by overproduction of the eEF1B subunits are unknown, this suggestion looks natural for the *sup35 *mutants, since it presumes that eEF1B acts as a GEF for eRF3. However, an overdose of the eEF1Bα can also suppress the mutational defect of eRF1. The suppressible *sup45-sl23*^*ts *^mutation causes amino acid replacement in the eRF1 N-terminal domain which is not implicated in interaction with eRF3. In agreement with this, *sup45-sl23*^*ts *^did not noticeably influence interaction between eRF1 and eRF3 [20]. Therefore, one can suggest that this mutation interferes with the ability of eRF1 to inhibit dissociation of the eRF3·GTP complex. This should decrease the overall levels of the ternary eRF1·eRF3·GTP complex, the abundance of which could be restored by the eEF1B-mediated intensification of exchange of GDP for GTP on eRF3.

## Conclusion

The data presented suggest that eEF1B, the nucleotide exchange factor of eEF1A, also catalyzes GDP for GTP exchange on eRF3, a GTPase, the C domain of which is structurally similar to eEF1A [2,3,7]. This reaction is required for eRF3 function both in translation termination and outside of termination. However, it is obvious that in yeast the eEF1B-mediated exchange of nucleotides is more critical for eEF1A than for eRF3. This follows from the observations that while eEF1Bα is normally essential for viability, cells can survive without the protein in the presence of excess eEF1A [49]. One can suppose that the requirement for GEF is less pronounced for eRF3 than for eEF1A, because of a unique property of eRF1 to increase affinity of eRF3 to GTP. Indeed, interaction with eRF1 stabilizes eRF3 in a GTP-bound form after spontaneous exchange of GDP for GTP [12-14]. No such inhibitor of GTP dissociation is known for eEF1A.

Structural similarity suggests that eRF3 has evolved from eEF1A. Our genetic data suggest that the accessory protein, eEF1B, stimulating the activity of elongation factor eEF1A preserved this ability for the termination factor eRF3 as well. Direct biochemical studies are necessary to confirm the role of eEF1B in nucleotide exchange on eRF3.

## Authors' contributions

IV performed a screen for dosage suppressors of synthetic lethality between the *sup45 *and *SUP35-C *mutations and participated in molecular genetic studies. GF and ES carried out the molecular genetic studies. VS participated in planning of experiments and drafted the manuscript. MT-A designed the study and wrote the manuscript. All authors read and approved the final manuscript.
